# Impact on peri-implant connective tissue of laser treated versus traditional healing abutments: a human clinical trials

**DOI:** 10.1186/s12903-023-03148-y

**Published:** 2023-06-27

**Authors:** Giulia Gaggi, Andrea Di Credico, Gianmaria D’Addazio, Barbara Ghinassi, Giulio Argentieri, Sergio Caputi, Angela Di Baldassarre, Bruna Sinjari

**Affiliations:** 1 Human Anatomy and Cell Differentiation Lab, Department of Medicine and Aging Sciences, University “G.d’Annunzio” of Chieti-Pescara, 66100 Chieti, Italy; 2 Unit of Prosthodontics, Department of Innovative Technologies in Medicine and Dentistry, University “G. d’Annunzio” of Chieti-Pescara, 66100 Chieti, Italy; 3 Electron Microscopy Laboratory, University “G. d’Annunzio” of Chieti-Pescara, 66100 Chieti, Italy

**Keywords:** Laser-treated surface, Peri-implant connective tissue, Gingiva, Tenascin C, Collagen, Fibrillin I, MMPs, TIMPs, Healing abutment, Peri-implantitis

## Abstract

**Background:**

Dental implant is the principal treatment for edentulism and the healthiness of the peri-implant tissue has a pivotal role for its longterm success. In addition, it has been shown that also the topography of the healing abutment can influence the outcome of the restoration. The objective of this human clinical trial was to assess the impact of a novel laser-treated healing abutment on peri-implant connective tissue and extracellular matrix proteins compared to the conventional machined surface, which served as the control group.

**Methods:**

During second surgical stage a customized healing abutment were inserted on 30 single dental implants. Healing abutments were realized with two alternated different surface (two side laser-treated surfaces and two side machined surfaces) in order to be considered both as test and control on the same implant and reduce positioning bias. Following the soft tissue healing period (30 ± 7 days) a 5 mm circular biopsy was retrieved. Immuno-histochemical and quantitative real-time PCR (qPCR) analyses were performed on Collagen, Tenascin C, Fibrillin I, Metalloproteinases (MMPs) and their inhibitor (TIMPs). 15 were processed for qPCR, while the other 15 were processed for immunohistochemical analysis. Paired t-test between the two groups were performed. A value of *p* < 0.05 was considered statistically significant.

**Results:**

Results revealed that the connective tissue facing the laser-treated surface expressed statistically significant lower amount of MMPs (*p* < 0.05) and higher level of TIMPs 3 (*p* < 0.05), compared to the tissue surrounding the machined implant, which, in turn expressed also altered level of extracellular matrix protein (Tenascin C, Fibrillin I (*p* < 0.05)) and Collagen V, that are known to be altered also in peri-implantitis.

**Conclusions:**

In conclusion, the laser-treated surface holds promise in positively influencing wound healing of peri-implant connective tissue. Results demonstrated that topographic nature of the healing abutments can positively influence mucosal wound healing and molecular expression. Previous studies have been demonstrated how laser treatment can rightly influence integrity and functionality of the gingiva epithelium and cell adhesion. Regarding connective tissue different molecular expression demonstrated a different inflammatory pattern between laser treated or machined surfaces where laser treated showed better response. Targeted interventions and preventive measures on peri- implant topography could effectively minimize the risk of peri-implant diseases contributing to the long-term success and durability of restoration. However, new studies are mandatory to better understand this phenomenon and the role of this surface in the peri-implantitis process.

**Trial registration:**

This trial is registered with ClinicalTrials.gov Identifier: (Registration Number: NCT05754970). Registered 06/03/2023, retrospectively registered.

## Background

The success of dental implants relies heavily on the health of the peri-implant mucosa, which plays a crucial role in protecting and maintaining restorations. Inflammation or infection of the soft tissues can lead to the loss of surrounding bone, ultimately resulting in implant failure [1].

Over the years, microscopic analysis and studies of soft tissues around teeth and implants has significantly improved the prognosis of dental and implant-prosthetic restorations [1, 2].

The major components of gingival connective tissue surrounding teeth and implant are the collagen fibers, that form a dense network close the root of the teeth. Type I and III collagens are the most prevalent type, found in the all the layers of the gingival connective tissue [3]. Additionally type V collagen is predominantly expressed in the basement membrane and blood vessels suppling the gingival mucosa. Type I collagen fibers are bound and stabilized by another abundant protein in the extracellular matrix, the Tenascin C. It has been reported that it is highly upregulated upon tissue damage and inflammation, but it is rapidly cleaved when the tissue is completely repaired [4]. Tenascin C also regulated various cellular responses such as cell migration, proliferation, attachment and can also modulate the activity of proteases, thereby influencing extracellular matrix degradation [5]. Fibrillin I is the primary component of the elastic fibers in the gingival connective tissue [6]. Although Fibrillin I is primarily a structural protein that helps maintain tissue architecture, it has been shown that Fibrillin-rich microfibrils also contribute to the regulation of Transforming Growth Factor-β (TGF-β) signaling. These microfibrils can bind TGF-β, controlling its storage and activation [7]. Indeed the degradation of Fibrillin I by matrix metalloproteinases (MMPs) is the most common mechanism to induced the release of TGF- β [8].

During the wound healing process, connective tissue should undergo a remodeling process coordinated by the activation of MMPs, which are upregulated by pro-inflammatory molecules [9]. MMPs are inhibited by endogenous proteins named Tissue inhibitors of metalloproteinases (TIMPs), which suppress the activity of MMP family members in a non-specific way [10].

These processes, involving MMPs and TIMPs, are present in both healing and pathological processes. Resolution of inflammatory processes during the healing of surgical wounds is necessary to ensure a healthy state around teeth and implants and to prevent the onset of pathologies. Peri-implantitis, one of the major complications affecting implant-prosthetic restorations, has been described as a destructive inflammatory lesion that affects soft and hard tissues, leading to bone loss and eventual implant failure [11–14]. The prevalence of peri-implantitis varies widely, with different studies reporting rates ranging from 14.38% to 36.6% [15, 16].

The 2017 World Workshop on the Classification of Periodontal and Peri-Implant Diseases and Conditions [17] proposed a new classification of periodontal and peri-implant diseases, where the diagnosis of peri-implantitis was a combination of probing depth ≥ 6 mm, loss of supporting bone ≥ 3 mm and the presence of bleeding on probing (BOP) and/or suppuration [17]. Following this classification, Diaz et al. in 2022 stated that Prevalence of peri-implantitis was 19.53% (95% CI 12.87–26.19) at the patient-level, and 12.53% (95% CI 11.67–13.39) at the implant-level and it remains highly variable even following restriction to the clinical case definition [18].

Regardless of whether it is a multifactorial disease, several studies have analyzed the relationship between biofilm and bone loss and investigated the influence of the physico-chemical properties of abutment materials on the success/failure of the implant [19, 20]. The successful integration of dental implants in the oral cavity relies on the resolution of inflammation and the health of the peri-implant connective tissue. The latter serves as the primary barrier against oral cavity pathogens and protects the surrounding alveolar bone and tooth roots [21–23]. Therefore, increased attention is being paid to implant material features, such as surface topography and chemistry, abutment micro and macro design and presence of biological proteins, which are known to influence the attachment of epithelial and connective tissue to the abutment surface [24].

Research has demonstrated that cell differentiation, protein film composition, and molecule absorption can be affected by surface treatment and the resulting differences in roughness [25, 26]. Studies have also found that the surface microtexture plays a role in the development of a more extensive fibrin scaffold around titanium, which has a positive impact on osseointegration and the formation of early connective tissue [27]. The Synthegra surface is a novel technology used for treating titanium surfaces [27, 28], and it has the potential to provide benefits in terms of bone responses and microbiological behavior. The laser-treated implant surface, which has nanopores with a diameter of 5 μm, a depth of 5 μm, and an interpore distance of 15 μm, has been investigated in various fields. In particular, in terms of osteoblast proliferation and adhesion, the laser-treated surface may be responsible for promoting cell growth [26]. Additionally, a separate study confirmed the effectiveness of the laser treatment in preventing bacterial proliferation, as it significantly reduced P. gingivalis biofilm formation [28].

Moreover, in previous studies we have evaluated the response of gingival epithelial tissue and the expression of pro-inflammatory molecules to a healing abutment characterized by a laser-treated surface. It was also demonstrated that the gingival epithelium facing the laser-treated surface expressed more adhesion molecules and lower level of inflammatory mediator [24, 25].

For this reasons, here we aimed to investigate the effect of the laser-treated surface on peri-implant connective tissue and extracellular matrix protein in comparison with the machined surface healing abutment, used as control.

## Methods

### Patient selection

This study was approved by the Ethical Committee of "G.d'Annunzio" Chieti-Pescara University on October 18, 2018, under the number 22. A total of 38 patients were recruited for the trial, comprising 25 men and 13 women with an average age of 56.5 ± 9.9 years. However, due to eight patients not meeting the inclusion criteria and lacking sufficient soft tissue quantity for biopsies, only a total of 30 patients were included in the study. The patients were from the Implantology Operative Unit of the Department of Medical, Oral, and Biotechnological Sciences at the University "G.d'Annunzio" of Chieti-Pescara. They gave written informed consent and received information about the study protocol. The study was conducted in accordance with the principles of the Helsinki Declaration established by the World Medical Association regarding human subjects. Briefly, inclusion criteria provide patients between 18 and 75 years old with good systemic and oral health and sufficient bone and soft tissue quantity. Specifically, the minimum criteria were a residual bone quantity of at least 1 mm following implant insertion, as described by Buser et al. in 2004 [29]. As for the soft tissues, a thickness was calculated to ensure a minimum of 2 mm of keratinized tissue (KT) after gingival punch as previously described [25] and described by Ravidà et al. in 2022 [30]. On the other hand, patients with active periodontal disease or poor oral hygiene evaluated by Full Mouth Plaque Score (FMPS) and Full Mouth Bleeding Score (FMBS) more than 25% at every stage, uncontrolled diabetes mellitus, and smoking more than 10 cigarettes per day were excluded.

### Surgical treatment

Surgical treatment has been thoroughly previously described [24, 25]. Briefly, healing abutments utilized in this study were composed of grade 5, whereas the implant fixtures were composed of commercially pure Titanium grade 4. Prior to implant placement, all patients underwent a thorough clinical and radiographic oral evaluation. A single surgeon performed the implant insertion through a full thickness flap. The implants were inserted using a two-stage protocol and were manufactured by Omny, Geass s.r.l, Pozzuoli del Friuli, Udine, Italy, with lengths ranging from 7.0 to 11.5 mm and diameters from 3.50 to 4.1 mm. Prior to the implant placement, all patients received a prophylaxis therapy (2 g/day for six days, Augmentin®, GLaxo-Smithkline Beecham, Brentford, UK) and were instructed to oral hygiene. All healing abutments were placed at T1 (12 ± 4 weeks) after evaluation of soft tissue inflammation. The evaluation of the absence of inflammation was conducted using both radiological and clinical assessments. From a clinical standpoint, the integrity of the soft tissues surrounding the implant site was examined to identify any indications of inflammation, including the absence of bleeding and/or fistulas in the area where the implant was inserted. In addition, radiological evaluations were performed to confirm the absence of peri-implant radiolucency or any other radiographic signs that could suggest inflammation. The combination of these clinical and radiological assessments allowed for a comprehensive evaluation of the absence of inflammation, ensuring that the placement of the healing abutments could proceed under optimal conditions. Each healing abutment was designed with two alternated surfaces: laser-treated/machined and machined/laser-treated. Moreover, customized healing abutment had an outer diameter of 2.65 mm. This has already been described in the previous study [25]. The reason for the reduced diameter was to minimize the size of the gingival punch. It was also considered that the presence of varying amounts of residual soft tissue, different oral hygiene condition, different soft tissue height, could potentially influence the analysis of results. This design allowed us to analyze the gingival response to the two different surfaces in the same patient, avoiding bias due to inter-subject variability. The customized healing abutment was removed after 30 ± 7 days with the soft tissue biopsy.

### Specimen retrieval and analyses

Following the healing period (30 ± 7 days) a circular sections (with a diameter of 5 mm) of the surrounding soft tissue were retrieved by the surgeon (G.D’A.) for analysis, as previously described [25]. A total of 30 samples were collected: 15 were processed for quantitative real-time PCR (qPCR), while the other 15 were processed for immunohistochemical analysis. The examiners were not informed about which areas of the tissue were in contact with the laser-treated surface versus the smooth surface of the abutments. The examiner was kept blind to this information throughout the evaluation process. To ensure consistency and accurate assessment, the tissue samples were marked with sutures and photographed by the surgeon who performed the procedures. The surgeon, being aware of the exact orientation, took precautions to maintain blinding during the examination phase. This approach was implemented to minimize bias and ensure impartial evaluation of the samples.

### RNA extraction and reverse transcription

Total RNA was extracted from gingival tissue biopsies using QIAzol lysis reagent (QIAGEN Hilden Germany) according to the manufacturers method. RNA concentration was measured with a Qubit 3 (Thermo Fisher Scientific Waltham MA USA). For reverse transcription 1 μg of RNA was retrotranscribed using the High Capacity cDNA Reverse Transcription Kit (Thermo Fisher Scientific Waltham MA USA) according to the manufacturers method.

### Quantitative real time PCR (qPCR)

For all the examined mRNAs, qPCR analysis was performed using SYBR green (PowerUp SYBR Green Master mix, Thermo Fisher Scientific, Waltham, MA, USA) as previously reported [31]. Each gene expression value was normalized to the expression level of 18S. The fold changes were obtained by ΔΔCt methods, using the gingiva from healthy donor as a control condition. The sequences of human primers used in the study were generated by Primer Blast unless otherwise indicated and are listed in Table 1.

**Table 1:** Primer sequences

Gene name	Primer sequence (5’à3’)
Collagen I_ FW	AACCAAGGCTGCAACCTGGA
Collagen I_ RV	GGCTGAGTAGGGTACACGCAGG
Collagen III _FW	CTCCTGGGATTAATGGTAGT
Collagen III_RV	CCAGGAGCTCCAGGAAT
Collagen V_FW	TGCTGAAAAAGGGGGTTTGC
Collagen V RV	TGTGGGTTCTCCTGAGAGTGA
Fibrillin I_FW	AGGAAACGGAGAAGCACAA
Fibrillin I_RV	CTGTCTTCTCAACATCCCAA
Tenascin C_FW	CAACCTGATGGGGAGATATGGGGA
Tenascin C_RV	GAGTGTTCGTGGCCCTTCCAG
MMP1 _FW	GCTAACAAATACTGGAGGTATGATG
MMP1_RV	GTCATGTGCTATCATTTTGGGA
MMP3_ FW	ATGATGAACAATGGACAAAGGA
MMP3_RV	GAGTGAAAGAGACCCAGGGA
MMP9 _FW [24]	CGCAGACATCGTCATCCAGT
MMP9 _RW [24]	GGACCACAACTCGTCATCGT
MMP13_ FW	CAGGAATTGGTGATAAAGTAGAT
MMP13_RV	CTGTATTCAAACTGTATGGGTC
TIMP1 FW [24]	CTGTTGGCTGTGAGGAATGC
TIMP1 RW [24]	CGGGACTGGAAGCCCTTTTC
TIMP2_FW	ACATTTATGGCAACCCTATCAA
TIMP2_RV	TCAGGCCCTTTGAACATCTTTA
TIMP3_FW	AGGACGCCTTCTGCAAC
TIMP3_RV	CTCCTTTACCAGCTTCTTCC
18S FW [32]	CATGGCCGTTCTTAGTTGGT
18S RW [32]	CGCTGAGCCAGTCAGTGTAG

### Immunohistochemical analyses

The gingiva samples were fixed in 10% neutral buffered formalin and then embedded to paraffin, identifying the point of passage between the region of the gingiva adherent to the laser-conditioned and the one adherent to the machined surfaces. 3-μm-sized paraffin-embedded tissue sections, were deparaffinized, washed and blocked and then subjected to antigen retrieval, using 10 mM sodium citrate for 20 min at 60 °C.

To evidence the collagen organization, the gingiva was stained by Trichrome (Bio-Optica, Milan, Italy) following the manufacturer’s procedures.

Immunohistochemistry was performed as previously reported [25] using the Mouse- and Rabbit-specific HRP/DAB (ABC) detection IHC kit (Abcam, Cambridge, UK) following the manufacturer’s instructions. Briefly, samples were then incubated with 1:50 anti-human Collagen V (Abcam), 1:50 Tenascin C 1: 50 MMP1, 1:50 MMP3 and 1:50 MMP13 antibodies (all from Thermofisher) overnight at 4 °C. Successively, slides were incubated with goat anti-polyvalent antibody for 10 min, and subsequently with peroxidase for 10 min. After incubation, samples were washed and treated by DAB substrate. At the end of the process, slides were counterstained with hematoxylin. Histological observations were carried out using a Evos M7000 (Thermo Fisher) Three randomly non-overlapping areas were chosen per each section.

### Statistical analysis

For the statistical calculation of the sample size in the present study, the statistical software Gpower sample size calculation was used [33], Specifically, for the sample size calculation in the comparison of two means on the same sample, a two-tailed t-test with α = 0.05 and power (1-β) = 0.95 and effect size d = 0.8 was used, resulting in a required sample size of 23 healing screws. Accounting for a 30% dropout rate, a total of 30 healing screws are necessary [34]. Each patient was considered as a single statistical unit. Therefore, for this type of analysis, only one biopsy was performed on each patient.

A paired t-test using GraphPad Prism 4.0 was used to perform the statistical analysis between the two groups. All data are presented as mean ± standard deviation (SD); a value of *p* < 0.05 was considered statistically significant.

## Results

**Fig. 1:**
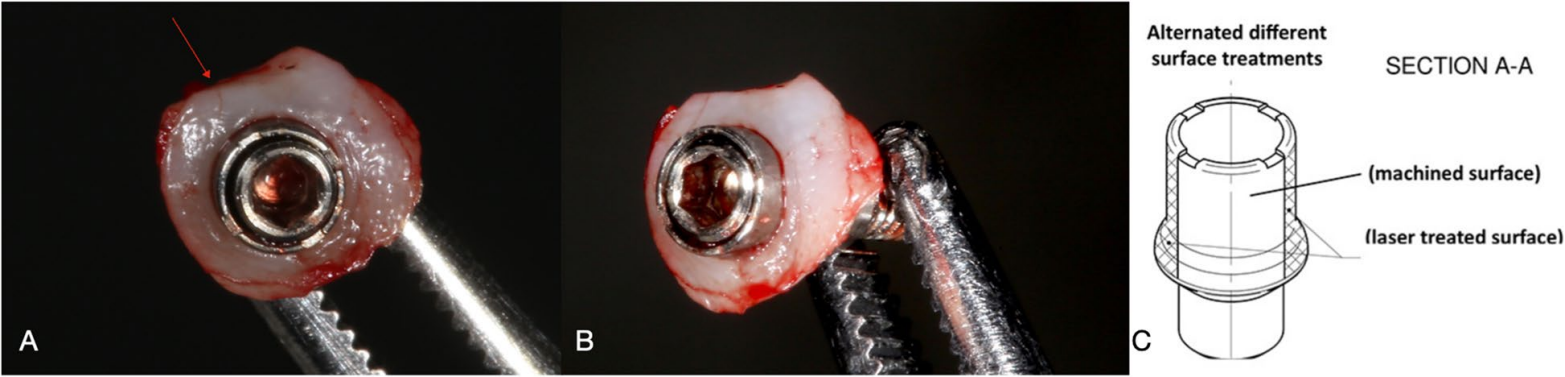
Explanatory images: **A** Occlusal vision of removal soft tissue and healing abutment. Arrow indicates micro-incision performed to know the spatial orientation of biopsy; **B** Soft tissue surrounding the experimental healing abutment; **C** technical design of experimental healing abutment

In this study a new experimental laser-treated/machined healing abutment was used. Each healing abutment was treated with two alternated different surface treatments (machined and laser treated surface) where the two surfaces were repeated with the following order: Laser treated/machined/laser treated/machined as shown in Fig. 1.

**Fig. 2:**
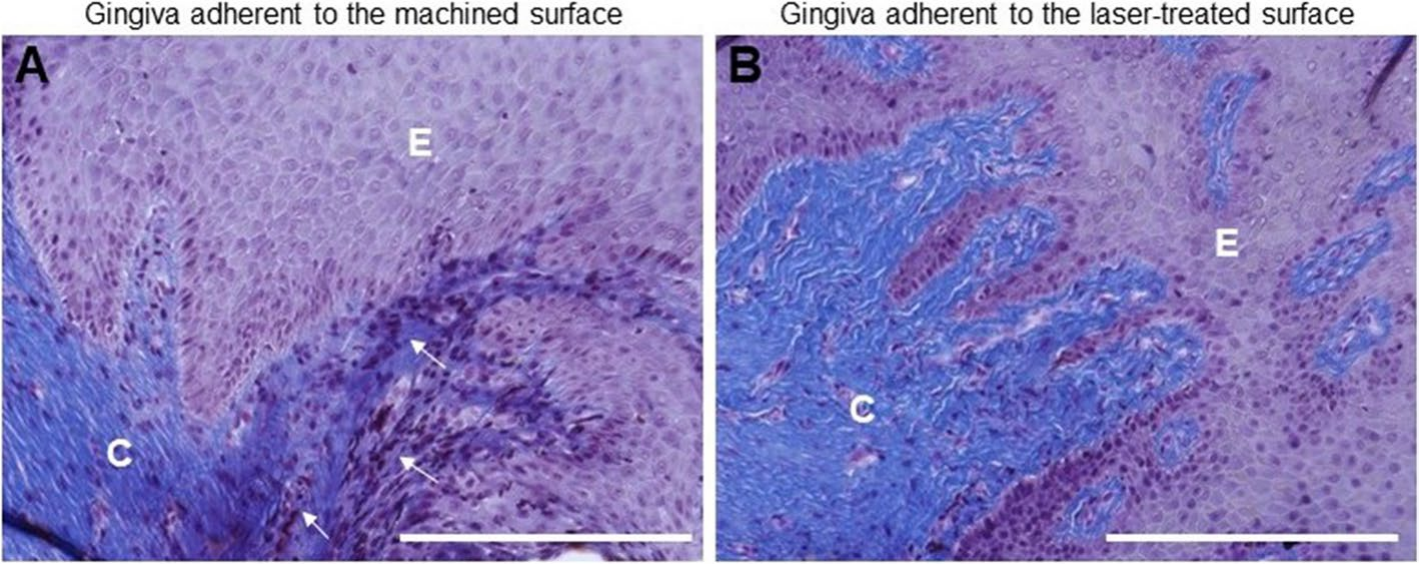
Trichrome staining of the region adherent to the (**a**) machined and (**b**) laser-treated surface. White arrows indicated the infiltration by mononuclear cells. Magnification 20x, scale bar 150 μm. The pictures are representative of 3 different randomly chosen fields E: epithelium, C: connective tissue

To investigate the impact of healing abutment on connective tissue, it was initially conducted an optical microscopic evaluation following trichrome staining. Results showed that the basal membrane of the tissue adhering to the machined surface was disrupted in certain areas, as depicted in Fig. 2A. Additionally, an inflamed area was detected in the connective tissue. On the contrary the gingiva facing the laser-treated surface showed a continued basal lamina without any inflammatory infiltrate (Fig. 2B).

**Fig. 3:**
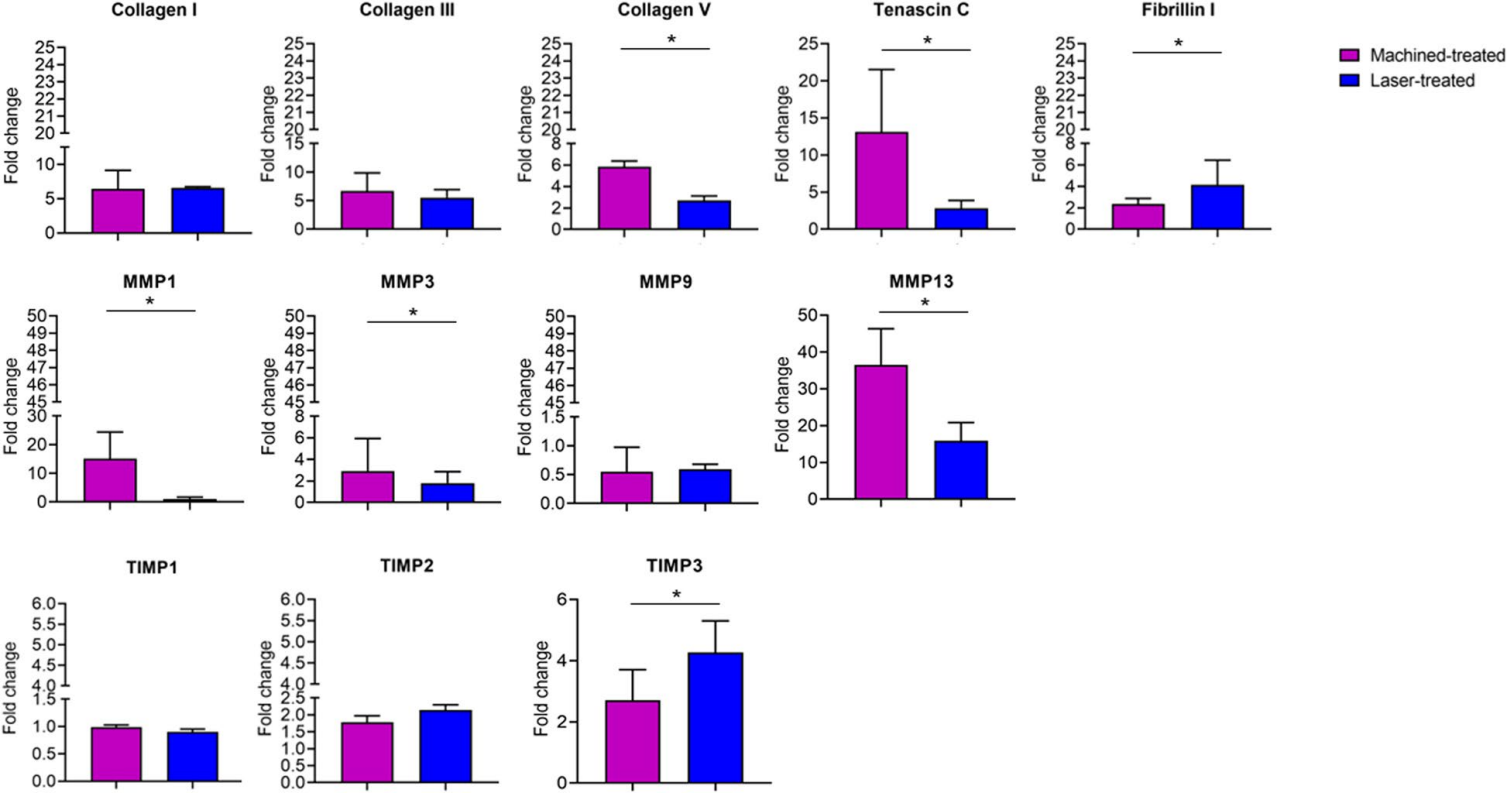
Gene expression of Collagen I, Collagen III, Collagen V, Tenascin C, Fibrillin I, MMP1, MMP3, MMP9, MMP13, TIMP1, TIMP2, TIMP3, as indicated in the gingiva facing the machined- and the laser- treated surface. Data are shown as fold changes (± SD) with respect to healthy donors (Fold change = 1). 18S was used as reference gene.​

Since the collagen I, III and V are the major component of the gingival extracellular matrix we also investigated whether the nanopored laser-treated surface and the classical machined one could affect collagen expression in the peri-implant tissue. Data obtained by qPCR showed a decrease of collagen V (fold change 5.8 ± 0.54 vs 2.7 ± 0.41 for machined and laser-treated surface, respectively, *p* < 0.05) in the gingiva facing the laser-treated surface, whereas no differences were detected in the expression of collagen I and III (Fig. 3).

**Fig. 4:**
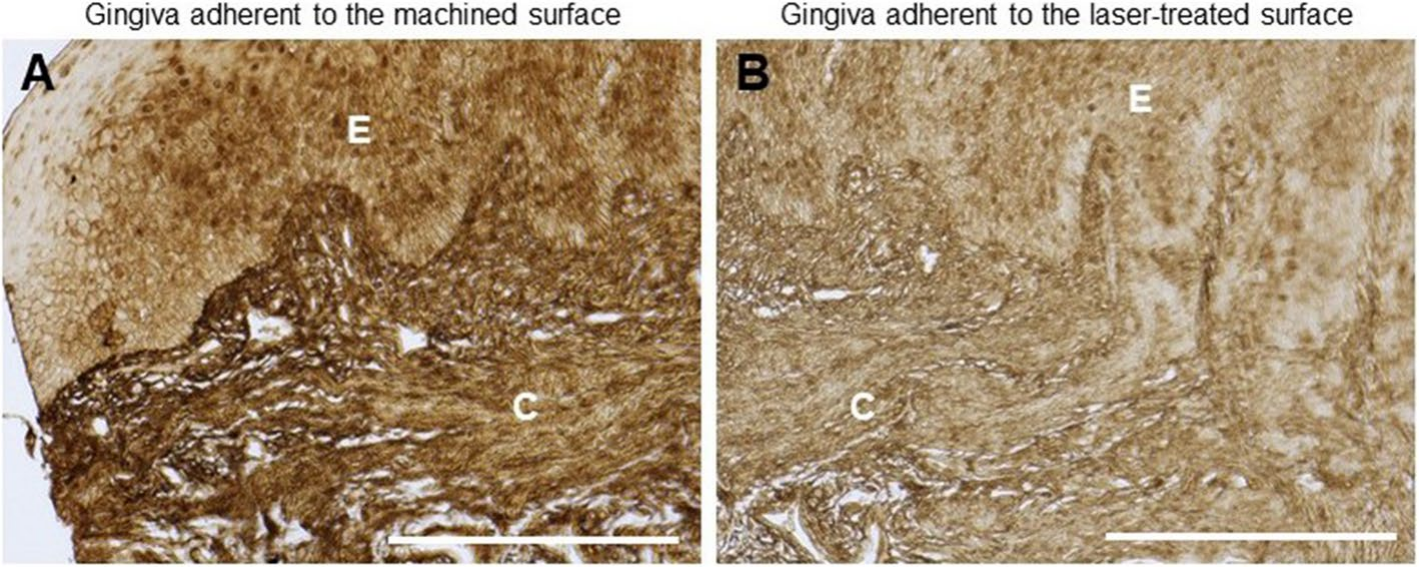
Immunostaining against Collagen V in the gingiva facing the (**A**) machined and (**B**) laser-treated surface​. Magnification 20x, scale bar 150 μm. The picture is representative of 3 different randomly chosen fields E: epithelium, C: connective tissue​

These data were also confirmed by immunohistochemical analyses showing a clear reduction of collagen V (% of positive area: 80 ± 12% vs 25 ± 4.5% for machined and laser-treated surface, respectively, *p* < 0.05) in the gingival tissue facing the laser-treated healing abutment (Fig. 4).

**Fig. 5:**
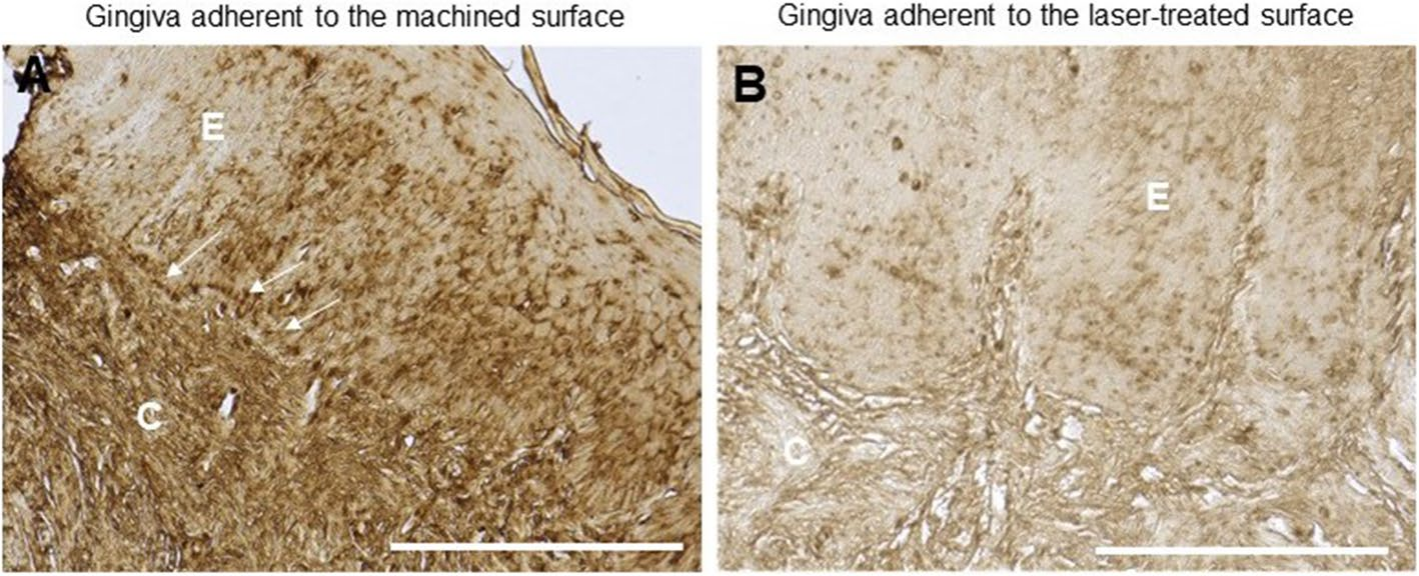
Immunohistochemical staining against Tenascin C in the (**A**) machined and (**B**) laser-treated surface. White arrows indicated the localization of Tenascin C at the basal membrane​. Magnification 20x, scale bar 150 μm. The pictures are representative of 3 different randomly chosen fields E: epithelium, C: connective tissue​

Tenascin C, usually upregulated in inflammatory conditions, resulted reduced in the peri-implant connective tissue facing the laser-treated abutment (fold change 13 ± 8.4 vs 2.8 ± 1.1 in machined and laser- treated surface, respectively, *p* < 0.05) (Figs. 3 and 5).

On the other side, the Fibrillin I,, was clearly increase in the laser treated-facing gingival (fold change 2.4 ± 0.52 vs 4.2 ± 2.3 in machined and laser- treated surface, respectively, *p* < 0.05) (Fig. 3).

**Fig. 6:**
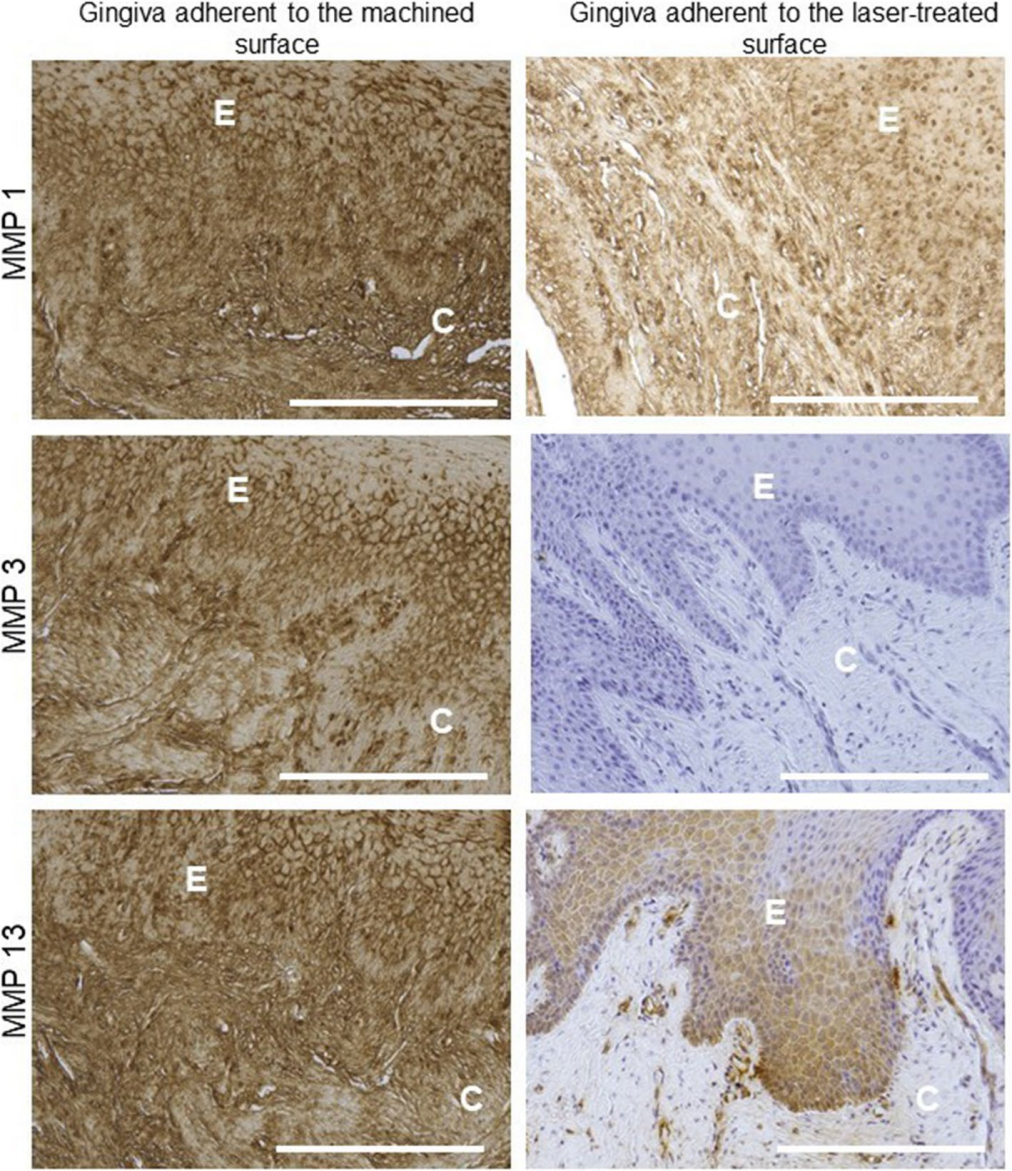
Immunohistochemical staining against MMP1, MMP3 and MMP13 in the machined and laser-treated surface (as indicated).​ Magnification 20x, scale bar 150 μm. The pictures are representative of 3 different randomly chosen fields E: epithelium, C: connective tissue​

Finally, we analyzed also the expression of MMPs and TIMPs in the tissues facing the machined and the laser treated surface, since both family proteins have a pivotal role in the connective tissue remodeling and tissue repairing,. Results demonstrated that MMP1 (fold change 15 ± 9.3 vs 1.1 ± 0.62 in machined and laser- treated surface, respectively, *p* < 0.05)., MMP3 (fold change 2.9 ± 3.0 vs 1.8 ± 1.1 in machined and laser- treated surface, respectively, *p* < 0.05) and MMP13 (fold change 37 ± 9.8 vs 16 ± 5.0 in machined and laser- treated surface, respectively, *p* < 0.05) expression was lower in the gingiva facing the laser-treated surface than the machined one, whereas no changes were detected in MMP9 transcript expression (Fig. 3). These data were also confirmed by immunohistochemical analyses, that showed a strong upregulation of MMP1 (% of positive area: 98 ± 15% vs 40 ± 12% for machined and laser-treated surface, respectively, *p* < 0.05) MMP3 (% of positive area: 90 ± 6.2% vs 5 ± 2.1% for machined and laser-treated surface, respectively, *p* < 0.05) and MMP13 (% of positive area: 95.3 ± 7.4% vs 15 ± 3.6 for machined and laser-treated surface, respectively, *p* < 0.05) in the gingival tissue adherent to the machined surface (Fig. 6).

On the other side, we detected an increase only in TIMP3 gene expression (fold change 2.7 vs 4.3 in machined and laser- treated surface, respectively, *p* < 0.05), whereas TIMP1 and 2 seemed to be not affected by the laser treated- or machined surface (Fig. 3).

## Discussion

The use of dental implant represents the the primary approach for addressing edentulism. However, complications such as reduced implant osseointegration and the onset of peri-implantitis can lead to implant failure and hinder oral functionality. Recent scientific investigations has shown that the micromorphology of the implant plays a significant role in determining the success or loss of the implant. As a result, there is a growing inclination to explore materials and abutment topography that enhance osseointegration while preventing bacterial growth and inflammation [35].

In our previous findings, we presenteted evidence indicating that the epithelial tissue facing the laser-treated surface expressed a higher level of adhesion molecules, while the expression of markers associated with inflammation was decreased [24, 25]. In this study, it was demonstrated that the connective tissue and extracellular protein expression also changed in response to the healing abutment surface. Specifically, in the connective tissue, we observed a decreased of Collagen V expression in the tissue facing the laser-treated surface, whikle there were no changes in the expression of Collagen I and III. Existing literature showed that a moderate increase of Collagen V occurs in peri-implantitis with a specific localization in the inflamed gingiva [10]. Conversely no differences in Collagen I and III expression were detected between healthy gingival and peri-implantitis, with some studies reported only changes on collagen fiber orientation [10, 36, 37].

Tenascin C is a prominent constituent of the extracellular matrix, with high levels of expression during embryogenesis. Interestingly, in the oral mucosa, Tenascin C continues to be expressed in adulthood, particularly in the papillary connective tissue located beneath the basement membrane. Its expression is closely associated with inflammation and rapid cell turnover.

 Indeed it is rapidly induced after tissue injury and the upregulation of Tenascin C mRNA often precedes signs of tissue damage and inflammation [38, 39], but it should decrease to basal level in the final stages of repair [40]. Our data showed a lower expression of Tenascin C at both mRNA and protein level in the gingiva adjacent to the laser-treated surface. Immunohistochemical analysis revealed that Tenascin C was distribuited in both the basal membrane and connective tissue in the peri-implant tissue facing the machined surface. This pattern indicates a a significant level of inflammation and infiltration by lymphocytes, as reported by Mane et al. [5]. These findings suggest that there is a reduced inflammation or accelerated healing in the peri-implant tissue surround the laser-treated surface compared to the one facing the machined healing abutment.

Similarity, Fibrillin I is one of the most abundant extracellular proteins found in microfibrils of connective tissue, which contributes to the limation of tissue elasticity. Since fibrillin-rich microfibrils bind the TGF-β, Fibrillin I insufficiency dysregulated the TGF-β signaling resulting in the up-regulation of tissue destruction-related genes such as metalloproteinases [41]. TGF-β, on the other hand, play a crucial role in regulating wound healing, extracellular matrix turnover, inflammation and angiogenesis. It also act also as a chemoattractant for neutrophils. Furthermore, an upregulation of TGF- β was found in case of failed implant [42] and periodontitis [43]. Previous reports have indicated that pro-inflammatory citokines can downregualate Fibrillin I expression and inadequate production of Fibrillin I has been associated with altered wound healing in the oral cavity of mice [41]. Our findings demonstrated a reduction in the expression of Fibrillin I in the peri-implant tissue facing the machined surface compared with the laser-treated healing abutment. This suggest the presence of a more pro-inflammatory environment and an impaired wound healing process. On the other side, it has been reported that the assessment of MMP levels in the gingival crevicular fluids during inflammation reflects the periodontal collagen metabolism and may have a diagnostic value. MMP1, 3, 9, 313 are the principal MMPs capable of degrading native collagen fibers in the inflamed human periodontium [9]. Since the MMP activity is regulated by the TIMPs, the integrity of connective tissue surrounding dental implant is influenced by a balance between MMPs and TIMPs [9, 10]. We observed an increased expression of all the MMPs analyzed (except the MMP9) in the tissue facing the machined surface both at transcript and protein level. Conversely, there was no upregulation of TIMPs in the gingiva surrounding the machined dental implant.

These data suggest an imbalance in the expression of MMPs and TIMPs, which may contribute to tissue destruction and elevate the risk of developing peri-implantitis. The opposite situation was found in the tissue facing the laser-treatment healing abutment, where we detected a lower expression of MMP1, 3 and 13 and an higher level of TIMP3. Two primary limitations of the study should be acknowledged. Firstly, the study was limited by the short duration of customized healing abutment placement in patients' mouths, which restricts the assessment of long-term outcomes. Although the focus was on evaluating the short-term effects of the customized healing abutment, it is crucial to recognize that comprehensive understanding of treatment efficacy and stability requires long-term observations. Secondly, the use of punch biopsies to analyze keratinized tissue introduced a certain level of invasiveness for the patients, despite efforts to minimize discomfort and complications. This invasive approach remains a study limitation. However, despite these limitations, the study's promising results offer valuable insights for future considerations. A noteworthy prospect would be to explore the application of this surface treatment to prosthetic components, aiming for improved soft tissue performance and long-term outcomes in the peri-implant area. By investigating the implementation of this superficial treatment on prosthetic components, enhanced long-term results and a more favorable peri-implant soft tissue environment may be anticipated.

## Conclusion

In conclusion, our findings suggest that the laser-treated surface holds promise in positively influencing wound healing of peri-implant tissue. This may potentially reduce the risk of peri-implantitis and implant failure, given the involvement of specific proteins in this biological phenomenon. However, it is important to acknowledge that peri-implant mucositis serves as a precursor to peri-implantitis. The strategies implemented on the abutment aim to mitigate bacterial colonization, enhance soft tissue healing, and prevent mucosal inflammation, specifically targeting mucositis. Therefore, further investigations are necessary to gain a deeper understanding of the impact of this surface on the peri-implantitis and mucositis process. In addition, a comprehensive understanding of the transmucosal portion will gradually lead to a significant reduction in the occurrence of peri-implant pathologies over time. By gaining in-depth knowledge of the factors influencing the transmucosal interface, such as the laser-treated surface discussed in this study, we can develop targeted interventions and preventive measures to effectively minimize the risk of peri-implant diseases. This will contribute to the long-term success and durability of dental implants, enhancing patient outcomes and overall oral health.

## Data Availability

The datasets used and/or analysed during the current study available from the corresponding author on reasonable request.
